# A Quantitative Approach to Mapping Mitochondrial Specialization and Plasticity

**DOI:** 10.21203/rs.3.rs-5961609/v1

**Published:** 2025-02-14

**Authors:** Martin Picard, Anna Monzel, Jack Devine, Darshana Kapri, Jose Enriquez, Caroline Trumpff

**Affiliations:** Columbia University Medical Center; Columbia University; Columbia University Medical Center; Columbia University Medical Center; Centro Nacional de Investigaciones Cardiovasculares; Columbia University Medical Center

**Keywords:** mitochondrion, multi-organ, cultured cells, computational, community resource

## Abstract

Mitochondria are a diverse family of organelles that specialize to accomplish complimentary functions 1–3. All mitochondria share general features, but not all mitochondria are created equal 4.Here we develop a quantitative pipeline to define the degree of molecular specialization among different mitochondrial phenotypes – or mitotypes. By distilling hundreds of validated mitochondrial genes/proteins into 149 biologically interpretable MitoPathway scores (MitoCarta 3.0 5) the simple mitotyping pipeline allows investigators to quantify and interpret mitochondrial diversity and plasticity from transcriptomics or proteomics data across a variety of natural and experimental contexts. We show that mouse and human multi-organ mitotypes segregate along two main axes of mitochondrial specialization, contrasting anabolic (liver) and catabolic (brain) tissues. In cultured primary human fibroblasts exhibiting robust time-dependent and treatment-induced metabolic plasticity 6–8, we demonstrate how the mitotype of a given cell type recalibrates i) over time in parallel with hallmarks of aging, and ii) in response to genetic, pharmacological, and metabolic perturbations. Investigators can now use MitotypeExplorer.org and the associated code to visualize, quantify and interpret the multivariate space of mitochondrial biology.

## Introduction

Biological organisms achieve efficiency and competency across a wide number of functions by developing specialized components. Organs form an interconnected network where muscles contract to seek and ingest food (supply), the gut digests and transports (assimilation), the heart beats and pumps (delivery), the liver transforms and synthesizes (anabolism), and the brain burns (catabolism) to integrate and signal ^9^. At the cellular level, a similar ecology of diverse units supports survival and adaptive capacity by expressing only a subset of the genetic potential in each cell, resulting in an array of specialized cell types and subtypes ^10,11^. Using omics techniques, hundreds of cell (sub)types can now be profiled based on their molecular signatures ^12,13^, yielding unprecedented insights into the stupendous biological complexity that confer cells and organs their unique metabolic properties. The rich ecosystem of omics-based profiling methods developed in the last decades has enabled transformative advances in cell biology. However, mitochondrial biology has somewhat lagged behind in relation to its nomenclature and approaches available to define mitochondrial specialization and plasticity ^4^.

**Figure 1:**
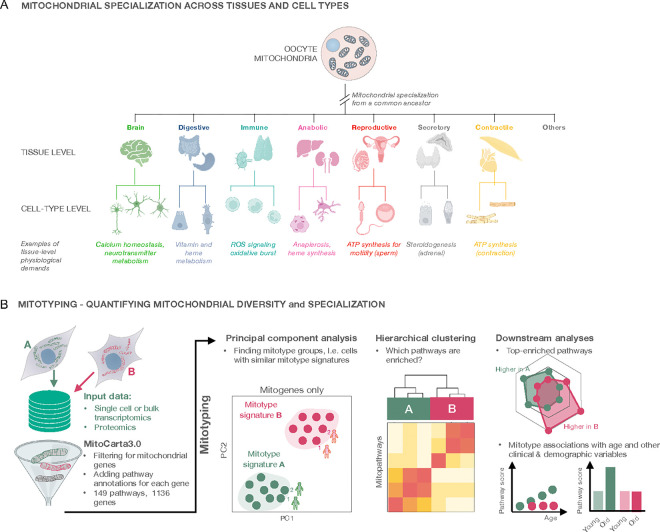
Quantifying mitochondrial specialization. **(A)** Mitochondrial specialization can be quantified at different levels of complexity. All mitochondria originate from the maternal mitochondrial population in the oocyte. During development, mitochondria must acquire tissue-specificity. Tissue-level mitotypes reflect mitochondrial specialization across the mammalian body and are a combination of multiple cell type-level mitotypes. Cells rely on specialized mitochondria that house a variety of pathways vital for cellular function. (**B)** Mitotyping is a method to quantify mitochondrial specialization from -omics data. Mitochondrial genes or proteins are extracted from normalized transcriptomics or proteomics data, and MitoPathway annotations are added using MitoCarta3.0 ^5^. In each tissue, cell type or cell state, the mitochondrial transcriptome or proteome is deeply characterized using MitoGenes or MitoPathways to find distinct mitotype signatures, identify groups with similar mitotypes or to study mitochondrial adaptation.

Much of a cell’s metabolic life involves the mitochondrion – a metabolic hub and signaling platform integrating numerous cellular and organismal behaviors ^2,14^. Like cell (sub)types, all mitochondria in the differentiated multi-organ and multi-cellular body arise from a single common ancestor, the oocyte (Figure 1A). The mitochondrial phenotype of human oocytes is rather unique ^15^. Oocyte mitochondria also are the “mother” of all terminally differentiated mitochondria types known to mitochondrial biologists – reflecting horizontal developmental plasticity. Different organs and cell types harbor specialized mitochondria ^16,17^, and different mitochondrial phenotypes are even observed among different sub-compartments of the same cell ^3,18–20^. Temporally, it is also established that exercise ^21,22^, nutritional interventions ^23^, hormones ^24^ and subjective experiences including psychosocial factors ^25–27^ induce specific functional recalibrations in mitochondrial biology, calling for a framework to quantify mitochondrial plasticity.

Most studies typically profile mitochondrial biology using one or a handful of molecular features, activities, or functional parameters. This can be misleading as a change in one or two parameters in isolation can appear to reflect “dysfunction”, whereas additional parameters that offer a more holistic picture can instead reveal adaptation e.g., ^28^. For example, if one function declines along with 100 other ones, it is more likely to reflect a decline in mitochondrial mass; but if the same function declines while a dozen other functions are selectively upregulated, it is more likely to reflect a meaningful recalibration pattern. Most techniques cannot differentiate between these possibilities, conflating recalibrations with “dysfunction” ^4^. Thus, a simple, scalable approach to generate interpretable and integrative indices of mitochondrial phenotypes would enable mitochondrial scientists to develop sensitive and specific interpretations of mitochondrial recalibrations under various experimental, physiological, and clinical contexts. The ability to quantify mitochondrial specialization based on proteomic or transcriptomic data could reveal distinct stable mitochondrial signatures, transient mitochondrial states, and/or patterns of mitochondrial plasticity that would otherwise remain inaccessible with conventional approaches.

Here we describe a simple computational approach to molecularly phenotypemammalian mitochondria (Figure 1B). To achieve this, we quantify the extent to which specialized mitochondria (i.e. mitotypes) molecularly diverge, and we determine in a given sample which *mitochondrial pathways* are prioritized. We term this approach *mitotyping*. As for cell types*,* a *mitotype* is defined by the set of over-expressed or prioritized mitochondrial pathways relative to other pathways in a given cell or tissue. We provide proof-of-concept examples illustrating how mitotyping expands our understanding of co-regulated and anti-correlated mitochondrial functions, tissue-specific metabolic phenotypes, key mitotype features distinguishing anabolic (liver) and catabolic (brain) tissues, and the plasticity of human mitochondria over weeks to months *in vitro*.

### Organ and tissue-based mitochondrial phenotyping

#### Mouse tissue-specific mitotypes

**Figure 2:**
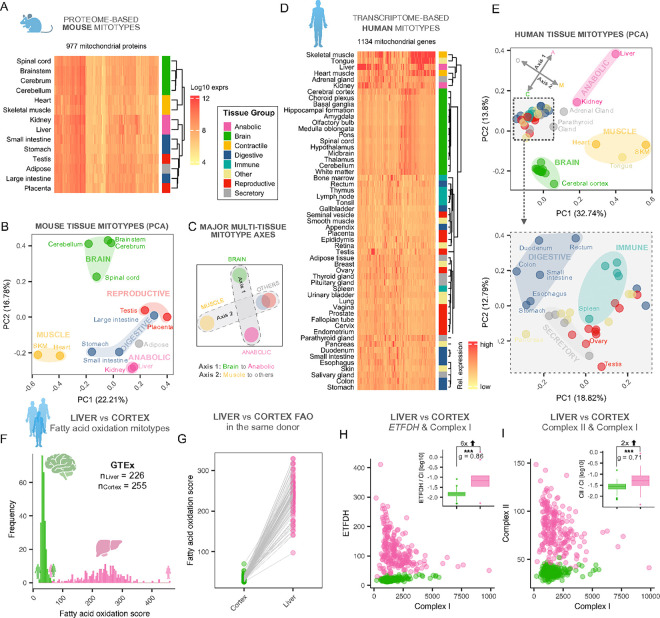
Mouse and human tissues contain molecularly distinct molecular mitochondrial phenotypes. **A)** Hierarchical clustering and **B)** Principal component analysis (PCA) of the 14 mouse mitochondrial proteomes from MitoCarta3.0 ^5^ reveals the grouping of physiologically-related tissues, based uniquely on mitochondrial proteins. **C)** Major multi-tissue mitotype axes as suggested by the PCA in B. **D)** Human mitotypes based on consensus gene expression data from the human protein atlas (HPA) and the Genotype-Tissue Expression (GTEx) project ^29,31^, mapped to the human MitoCarta3.0 gene list. Relative expression for each mitochondrial gene (columns) in 55 human tissues (rows), color-coded by physiological systems. **E)** PCA of all human mitotypes (top) and of the tissue subsets highlighted in the box (bottom), illustrating the mitotype-based clustering of tissues with similar physiological functions. The PCA shows the same major tissue axes as in C (arrows in top panel). Axis 1 separates the CNS from anabolic tissues, and axis 2 separates muscle from other tissues. **F)** Fatty acid oxidation (FAO) in tissues from axis 1 (cerebral cortex vs liver) across >220 human donors from the GTEx project showing higher expression of FAO in the liver relative to the cortex. **G)** FAO in donor-matching samples from cortex and liver showing that liver FAO score is on average 7.1x higher than in the cortex **H)** Bivariate plot of FADH-dependent electron transfer flavoprotein dehydrogenase *ETFDH* and Complex I (CI) expression score and boxplot of *ETFDH*/CI ratios in cerebral cortex vs liver. The liver expresses 6x more ETFDH/CI compared to the brain. **I)** Bivariate plot of Complex II (CII) and Complex I (CI) expression scores indicating higher expression of CII in the liver and similar expression of CI in liver and cortex. The ratio of CII / CI is twice as high in the liver relative to cortex. Abbreviations: *SKM*, skeletal muscle; Hierarchical clustering methods in A and D using the Euclidean distance and the Ward’s D2 hierarchical Agglomerative Clustering Method ^52^. Brunner Munzel test and Hedges’ g for effect size. ***p<0.001.

Using the original MitoCarta mouse proteome dataset ^16^, we first visualized mitochondrial protein levels (n=977 proteins) from 14 tissues (**Supplemental Figure S1A**). Hierarchical clustering of mitochondrial genes alone – *without* any canonical tissue-specific or cell-type specific marker – yielded clusters of tissues that matched broad functional categories such as brain, contractile muscles, anabolic tissues, and all other tissues (Figure 2A). A principal component analysis (PCA) illustrated particularly clearly how tissues from the brain exist as a distinct mitotype cluster, segregating away from the anabolic liver and kidney, and from the contractile mitotype signature of cardiac and skeletal muscles. A fourth group of mixed tissues included digestive, reproductive, and adipose tissue mitotypes (Figure 2B). This integrative analysis points to two main conclusions: First, mitochondrial proteins alone are sufficient to distinguish mouse tissues, revealing the robustness of the mitotype proteomic signatures. Second, the cardinal alignment of tissues along four poles suggested the presence of *two major axes* of mitochondrial specialization (Figure 2C).

#### Human tissue-specific mitotypes

We repeated this analysis using RNA transcripts abundance, again restricted only to mitochondrial genes (n=1,134) across 55 human tissues from the Human Protein Atlas (HPA) ^29,30^ and Genome-Tissue Expression (GTEx) ^31^ databases (a consensus dataset of both resources, **Supplemental Figure S1B**). As in mice, the unsupervised analysis replicated the clustering of brain, anabolic, and contractile tissues, and a mixed group of the digestive, reproductive, endocrine, and immune system tissues (Figure 2D). Here also, tissues aligned along the same two axes of mitochondrial specialization extended from the most anabolic of all organs, the liver, to the most catabolic, the brain (*Axis 1*); and from contractile muscle tissues (heart, muscle, tongue) to other tissues (*Axis 2*) (Figure 2E). Moreover, further analysis of “other” tissues by PCA showed remarkably coherent mitochondrial transcriptional signatures for most digestive tube segments, for most immune tissues (except the spleen), and for reproductive or secretory tissues.

#### Pathway level mitotyping

To grasp the magnitude of the molecular specialization between tissues, we examined the expression of the well-characterized MitoPathway fatty acid oxidation (FAO) in samples from women and men (n=226–255), comparing the brain known to have low-to-absent FAO capacity ^32^ to the liver that readily oxidizes lipids for oxidative phosphorylation (OxPhos) and anaplerosis ^33,34^ (**Supplemental Figure S1C, 2A-B**, Figure 2F). The mean difference in fatty acid oxidation score between these tissues was 6.7-fold (p<0.0001, **Supplemental Figure S2D**). Comparing FAO expression in the liver and cortex from the same donor, we show that as expected from the population-level data, the FAO score is *always* ~3–11x higher in the liver than in the brain (average=7.1-fold, Figure 2G), establishing the reproducibility and magnitude of differences in mitochondrial gene expression between these human tissues. This result illustrates both the quantitative differences between tissues, and the internal consistency of mitotype signatures across individuals.

#### Gene-pathway level mitotyping

To further investigate the connection between FAO and the mitochondrial electron transport chain (ETC) in both liver and brain tissues, we explored this relationship both at the gene and pathway levels. FAO delivers electrons to the ETC by oxidizing acyl-CoAs in the mitochondrial matrix via both the TCA cycle and the FADH-dependent electron transfer flavoprotein dehydrogenase *ETFDH*. The latter directly transfers electrons to Coenzyme Q (CoQ), bypassing complex I (CI). Consequently, during FAO, the proportion of total electrons entering CI is reduced. Thus, compared to brain mitochondria where FAO is limited, we expect liver mitochondria to express more *ETFDH* relative to CI. The brain/liver mitotype difference on a bi-variate mitotype space shows as predicted that compared to the brain, the liver mitotype expresses *ETFDH* at ~6 times the level of CI (p<0.001), highlighting the quantifiable tissue-specific adaptation in mitochondrial electron flow on the gene-pathway level.

#### Gene level mitotyping

Building on our pathway- and gene-pathway-level mitotyping analyses, we next focused on gene-level mitotyping to specifically examine enzymes that inject electrons into the ETC while bypassing CI. These include complex II enzymes (e.g., *SDHA-D*), dihydroorotate dehydrogenase (*DHODH*), choline dehydrogenase (*CHDH*), prolyl dehydrogenase (*PRODH1*), hydroxyproline dehydrogenase (*PRODH2*) and sulfide quinone oxidoreductase (*SQRDL*). We show that FAD-dependent enzymes are expressed at lower levels in brain compared to liver mitotypes (**Supplemental Figure S2D**). The only exception is PRODH, which is expressed at higher levels in the brain, potentially due to its involvement in glutamate synthesis ^35^. This observation prompted us to further evaluate mitotype adaptation to the branched electron entry points of the ETC. Consistent with our earlier findings, relative to CI expression, the liver mitotype is enriched for multiple FAD-dependent enzymes (**Supplemental Figure S2E)** and is also enriched for complex II by 2.1-fold (p<0.0001) (Figure 2I), confirming pathway-level patterns at the level of single genes.

### A scalable approach to computing MitoPathways

**Figure 3:**
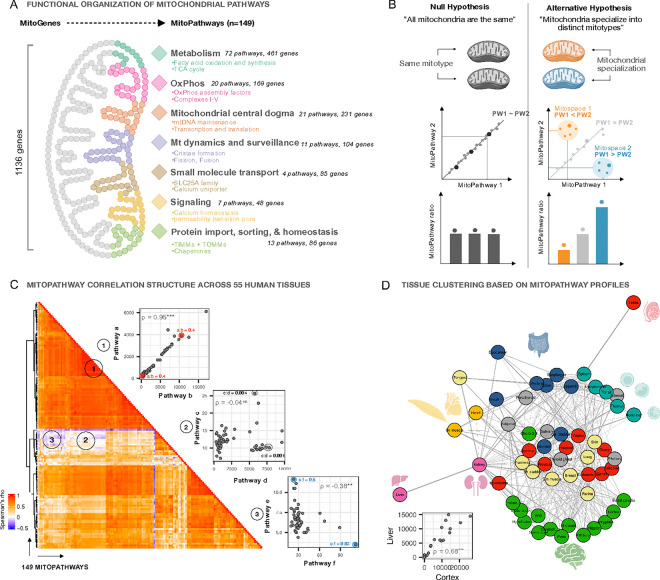
Quantifying mitochondrial specialization using MitoPathway scores. **A)** Mammalian mitochondria encompass >1100 genes and proteins, the majority of which are encoded in the nuclear genome and a small fraction (13 genes) in the mitochondrial genome. The expression of MitoGenes and MitoProteins varies in a context-dependent manner depending on the environment. Different sets of MitoGenes and MitoProteins form MitoPathways that are enriched in different tissues, cell types, or cell states. We use MitoPathway scores based on MitoCarta3.0 ^5^ annotations to quantify mitotype differences. **B)** Conceptual framework of mitochondrial specialization on the molecular level. *Left panel*: without mitochondrial specialization, all MitoPathways would display a proportional expression, and ratios of two MitoPathway scores would be the same. *Right panel*: Mitochondrial specialization is indicated by a tissue or cell occupying a distinct mitospace on a bivariate plot relative to other tissues or cells. Ratios of a set of two MitoPathways differ between the groups. **C)** Spearman’s r correlation-based MitoPathway analysis shows that not all MitoPathways are tightly correlated across 55 human tissues from the Human Protein Atlas dataset. On the right side of the heatmap are representative bivariate MitoPathway plots of 1) positively correlated, 2) not correlated, and 3) negatively correlated MitoPathways. In addition, numbers indicate MitoPathway ratios for some example tissues that occupy different mitospaces. **D)** Adjacency matrix presentation of between-tissue spearman correlations across 55 tissues and 149 MitoPathways demonstrating similar group clusters as on the gene level. The bivariate plot on the bottom shows a representative association between liver and cerebral cortex. **p<0.01, ***p<0.001.

Because the functions of many (albeit not all) mitochondrial proteins are known, tissue-specific mitotypes can be interrogated to gain insight into potential functional divergences that reflect the division of labor across tissue mitotypes. Some organs synthesize specific metabolites that are uniquely consumed by other organs, reflecting a network of metabolic interdependence ^36^. The expression of 149 MitoCarta 3.0 pathways ^5^ reflecting specific mitotype features, functions, and behaviors, can be computed as composite scores, or *MitoPathway scores* (Figure 3A). A MitoPathway score is calculated from the average expression of all genes within the pathway lists as defined in MitoCarta3.0 without modification (**Supplemental File 1**). Comparing the resulting scores between samples or tissues therefore quantifies the extent to which a given set of MitoPathways is expressed. Consequently, contrasting two MitoPathways on a bivariate plot across a given dataset shows which cells or tissues prioritize one pathway over the other. Those indices are quantifiable.

The null hypothesis – if all mitochondria were created equal and similarly specialized – is that an organ, tissue, or cell with more mitochondria than another sample would express higher levels of all MitoPathways in equal proportion (Figure 3B, *left*). Alternatively, if tissue mitochondria were specialized and expressed more of MitoPathway 2 relative to MitoPathway 1, or vice versa, they would occupy a distinct mitotype space relative to the null hypothesis (Figure 3B, *right*). The distinct liver and brain mitotype spaces with CII vs CI and FAO scores shown above demonstrate this point (see Figure 2I, Supplemental Figure S2G).

Examining the correlation structure of all 149 MitoPathways across 55 human tissues reveals a strong core of universal or “*canonical*” MitoPathways which are tightly correlated. If a tissue has more mitochondria, it has proportionally more of both canonical MitoPathways (*Example 1* in Figure 3C). However, some other combinations of MitoPathways are not correlated across human tissues (*Example 2*). And in some cases, MitoPathways were found to be negatively correlated (*Example 3*), indicating that if a given tissue or cell upregulates MitoPathway1, MitoPathway2 is downregulated. This could be driven by biological processes that would otherwise compete for the same substrate or co-factor, or by functions that are uniquely performed by only some specialized mitochondria. The clustering of tissues based on mitochondrial genes alone above (Figure 2A–E) refutes the null hypothesis (“all mitochondria are equal”) which predicts that tissues cluster randomly.

To further test this idea, we extended the multivariate analysis at the pathway level using tissue-to-tissue correlations with the 149 MitoCarta pathways as sole input. The network structure of all 55 tissue correlations illustrates distinct clusters of brain, anabolic, contractile, immune, and a mixed group of reproductive, secretory and other tissues (Figure 3D), similar to gene-level grouping. This suggests that vastly different human mitotypes representing families of organelles mapping onto the biology of their home tissue can be mapped by the combinations of the 149 interpretable MitoPathways.

### Multivariate mitotype specialization

To systematically describe and quantify multivariate patterns of mitochondrial specialization across multiple functional domains, we took two different approaches.

**Figure 4:**
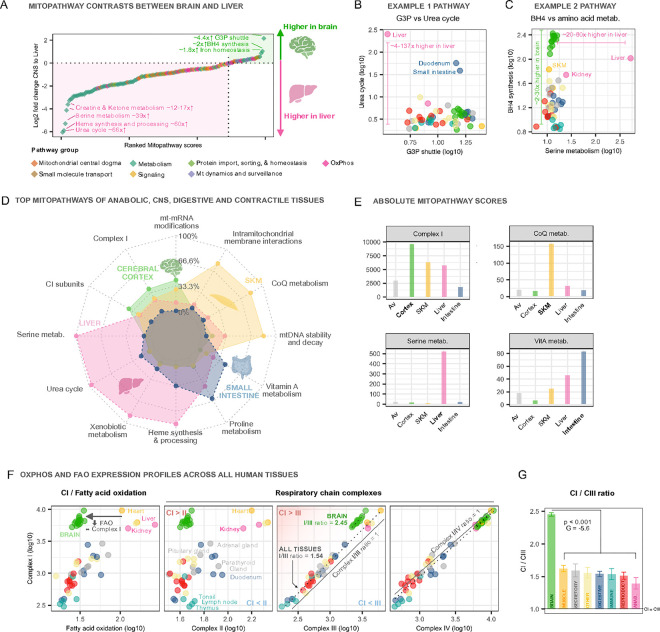
Comparing human MitoPathways between tissues. **A)** Rank ordering of all MitoPathway scores (n=149 Level 3 pathways, MitoCarta3.0) by the expression difference between brain (averaged across 14 brain tissues) and liver. Pathways are color-coded by major pathway groups (Level 1 pathways). Pathways with the greatest difference between liver (bottom) and brain tissues (top) were annotated. **B)**Urea cycle score vs Glycerol 3 phosphate (G3P) shuttle score across 55 human tissues, showing that liver mitochondria specialize in the Urea cycle while de-prioritizing the G3P shuttle. **C)**BH4 synthesis vs serine metabolism score showing that brain mitochondria specialize in BH4 synthesis, while liver mitochondria prioritize serine metabolism. **D)**MitoPathway radar chart of four tissues representing four major groups (brain, anabolic, contractile muscle, and digestive). For each tissue, MitoPathway scores were calculated, and the data was z-score-transformed within each pathway across the entire dataset. Top pathways for each of the four tissues were isolated (highest zscore across all tissues) and visualized. The boundaries of the radar chart are the highest (serine metabolism in liver, zscore = 7.3) and lowest (mt-mRNA modifications in small intestine, zscore = −0.8) values +/− 10%. **E)**Bar chart of absolute MitoPathway scores from top pathways shown in D. Although the absolute scores show which tissue dominates in comparison to the other tissues, absolute scores between two pathways should not be directly compared, as they can be confounded by multiple transcripts resulting from higher mitochondrial content (see methods for details). **F)**Bivariate plots of OxPhos and FAO expression profiles in the HPA dataset, illustrating the molecular specialization of mitochondria consistent with known physiological and functional characteristics of each tissue. The solid lines represent identity lines (x = y), the dotted lines indicate linear regression estimates for brain and all other tissues separately. **G)**Mean ratios (±SEM) of CI/CIII pathway scores across tissue groups. Welch’s t-test and hedges’ g for effect size. Abbreviations: G3P, Glycerol-3-phosphate; BH4, Tetrahydrobiopterin; metab., metabolism; SKM, skeletal muscle; CoQ, CoenzymeQ; VitA, Vitamin A; CI/II/III/IV, Complex I/II/III/IV; Anab., anabolic.

First, we contrasted all MitoPathways across brain and liver tissues, systematically asking which MitoPathways are more highly expressed over others, and by how much (Figure 4A). Two MitoPathway pairs higher in liver (relative to brain) or higher in brain (relative to liver) are visualized as MitoPathway scores in bivariate plots. Compared to all other tissues, the liver mitotype was enriched by 4–137-fold (48x on average) for *Urea cycle* (the liver is responsible for clearing the body’s ammonia by transforming it to urea ^33^). The brain was among other tissues enriched for *Glycerol-3-phosphate shuttle* (G3P, brings reducing equivalents from the cytoplasm to the mitochondrial electron transport chain ^37^) (Figure 4B), a shuttle that appears to be of low relevance to liver mitochondria. The second pair of most differentially expressed MitoPathways were *Tetrahydrobiopterin (BH4) synthesis* (2–30x higher in brain, used as co-factor for neurotransmitter synthesis ^38^) and *Serine metabolism* (20–80x higher in liver, used in anaplerosis and lipogenesis ^39^) (Figure 4C). These two-tissue systematic contrasts highlight mitochondrial molecular specialization related to known tissue physiology.

Our second approach consisted in examining the top enriched MitoPathways across the major axes of mitochondrial specialization (the four poles along the multi-tissue mitotype axes, see Figure 2C). Figure 4D illustrates families of MitoPathways coordinately enriched among the i) small intestine, ii) skeletal muscle, iii) liver, and iv) cerebral cortex. Each organ exhibited a coherent pattern of overexpression for a family of biologically-related metabolic pathways. In particular, the liver mitotype was expectedly specialized for multiple complimentary anabolic pathways (serine and urea metabolism, heme synthesis), as well as xenobiotic metabolism, in line with the liver’s role in systemic detoxification. In contrast, the skeletal muscle mitotype specialize in electron transport via Coenzyme Q, and the intestine mitotype in vitamin A and proline metabolism, and the brain in complex I-related biology (Figure 4E), possibly underlying its selective vulnerability to complex I-related disease ^40^. These largely confirmatory findings allow to visualize and quantify mitochondrial specialization.

Using this simple framework, we then specifically examined whether mitochondrial specialization occurs within a critical subsystem of mitochondrial biology: OxPhos. The OxPhos system is generally considered the core of mitochondrial biology as it produces and converts the electrochemical gradient into usable forms of energy such as ATP and NADPH ^4,14^. As tissue mitotypes exhibit higher FAO expression, they also express more of OxPhos complex I (CI), resulting in a roughly linear relationship between both MitoPathways. However, the brain stands out as expressing unusually high levels of *CI* relative to *FAO* (Figure 4F, *left*). In fact, even relative to OxPhos complex II (CII) and complex III (CIII), all brain regions express higher levels of CI (Figure 4F, *middle*). Interestingly, all tissues exhibited the same relative abundance of CI and CIV (Figure 4F, *right*). Mitotype ratios demonstrated that the brain expresses on average 60% more CI relative to CIII (p<0.001, Hedges’ g = −5.6). The CI:CIII ratio is 2.5 in the brain vs 1.5 on average across all other tissues (Figure 4G). In contrast, the average CI:CIV ratio in the brain is similar to all other tissues (CI:CIV = 0.9, **Supplemental Figure S4B**). Both the brain and heart overexpress CI relative to all other MitoPathways, supporting a particularly central role of CI and energy transformation in these tissues (**Supplemental Figures S4D,E**).

To summarize, so far we have showed how using two-tissue contrasts, multi-tissue and multi-pathway signatures, as well as MitoPathway ratios, confirm and quantify the magnitude of mitochondrial specialization across physiologically divergent organs and tissues.

### Mitochondrial pathway prioritization scores (mitoPPS)

While informative, the approach based on absolute MitoPathway scores described above is limited in three ways: **1)** It is influenced by overall mitochondrial gene expression level, meaning tissues with a higher mitochondrial content (kidney, heart, liver, muscle, brain) will always have the highest scores (lowest to highest scores scale across five orders of magnitude) than tissues with few mitochondria. **2)** It lacks directionality and does not sufficiently quantify “deprioritization”, such as the G3P shuttle in the liver (see Figure 4B). And **3)** MitoPathway scores are relative between samples across a given dataset (i.e. higher in tissue A relative to tissue B or to the average of other tissues/samples), and cannot be expressed and compared in absolute terms. This prevents comparisons across different datasets and modalities.

To resolve these limitations, we developed an approach that integrates MitoPathway *ratios* rather than absolute scores between all 149 pathways across 55 tissues. This produced ~1.2M comparisons and values scaling across eight orders of magnitude. To make the resulting metric independent of absolute expression levels and tissue mitochondrial content, we generated corrected ratio-based scores that quantify the extent to which each MitoPathway is prioritized in a given sample, relative to all other MitoPathways and samples. The resulting *mitochondrial Pathway Prioritization Score (mitoPPS)* and underlying computational structure is illustrated in **Supplemental Figure S5** with Complex I in brain and liver as example.

Briefly, the mitoPPS approach consists in normalizing each MitoPathway expression to each of the other 148. The resulting normalized ratios are then averaged for each pathway and tissue. This yields the fraction of the mitochondrial transcriptome devoted to each MitoPathway relative to other pathways. It thus reflects how much a given function or behavior is “prioritized”, or how much resources a given cell/tissue tries to invest in a given MitoPathway (Supplemental Figure S5A-B). The resulting simple linear scalar is an interpretable metric – the higher the score, the more prioritized is a given MitoPathway. The mitoPPS scores are also comparable across samples and datasets, so long as they provide similar coverage of the underlying genes/proteins. Importantly, the mitoPPS scores generated do not suffer from the overall mitochondrial abundance bias. The mitoPPS and the z-scored MitoPathway expression scores were significantly correlated, particularly when examining non-mtDNA (Spearman’s rho_non-mtDNA_=0.31, p<0.001) vs mtDNA-encoded MitoPathways rho_mtDNA_=0.73, p<0.001) separately (**Supplemental Figure S6**).

#### Quantifying absolute mitotype specialization with mitoPPS

**Figure 5:**
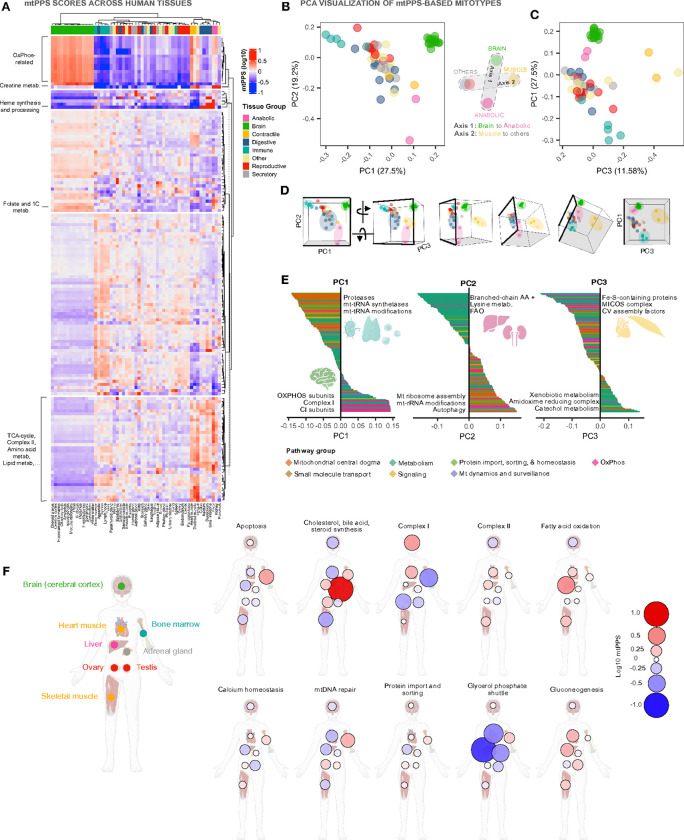
Tissue mitochondrial Pathway Priority Scores. **A)** Heatmap and hierarchical clustering of mitoPPS in log10 scale in the HPA dataset showing distinct prioritization signatures for different tissues, where a score of 0 represents average priority. Rows are pathways and columns are tissues. For instance, compared to average prioritization across all tissues, the brain prioritizes OxPhos-related pathways (except Complex II) by an extra ~110% (i.e., MitoPPS value = 2.1), while immune tissues deprioritize OxPhos by −65% (MitoPPS value = 0.35). Rows are k-means clustered, and column clusters are calculated using the Euclidean distance and the WardD.2 method. **B)** Principal component analysis of mitoPPS shows that PC1 separates immune and brain tissues, while PC2 separates anabolic tissues, and **C)** PC3 separates contractile muscle tissues. **D)**3D representation of PC1, 2, and 3. **E)**Loadings for PC1, 2, and 3. The top 3 pathways are highlighted, and the pathway loadings are color-coded by level1 MitoPathway annotations. Tissue symbols indicate the tissue group mostly associated with each PC. **F)** mitoPPS across the human body for a subset of 8 tissues, and 10 MitoPathways. mitoPPS on log10 scale, with 0 being average priority (white, small circle), 1 being higher priority (red, large circle), and −1 indicating low priority (blue, large circle). Abbreviations: PC, principal component; AA, amino acid

Applied to the multi-tissue dataset, the mitoPPS produces an interpretable metric of MitoPathway prioritization for all 55 tissues (Figure 5A). Compared to the raw MitoPathways scores, the dynamic range for mitoPPS scores is three orders of magnitude (~1,000-fold, ~0.01 – 14), likely a closer representation of the physiological dynamic range of mitochondrial specialization across the human body than the four order of magnitude observed for MitoPathway scores.

Some striking findings include: i) Brain tissues are most enriched for *OxPhos* whereas immune cells have the lowest *OxPhos* expression (6-fold difference, p<0.0001). ii) *Heme synthesis and processing* dominates in the liver, as well as some, albeit not all, digestive tissues (16-fold difference, p<0.0001). iii) *Creatine metabolism* is most prioritized in both anabolic and contractile muscle tissues, as well as pancreas (10-fold difference, p<0.001). and iv) *Folate and 1C metabolism* is most prioritized among brain tissues (except the choroid plexus) and liver (2.3-fold difference, p<0.0001). The quantitative distribution of mitoPPS scores emphasizes the interdependence of organ systems ^36^, borne out of the apparently exclusive specialization of mitochondria for some functions (discussed further below).

Using MitoPathway-based mitoPPS scores as input, we then again projected the data in a PCA space. The first three components of the model accounted for ~58% of the variance across tissues (Figure 5B–D). The brain mitotype again emerged as strikingly distinct, opposed by anabolic tissues and the liver in particular, muscles, and other tissues – similar to the results based on raw MitoPathway scores. Using the simplified PCA multivariate space, we then focused on the first three PCs and extracted the top MitoPathways that load (i.e., contribute) most strongly to each axis (Figure 5E, **Supplemental File 2**). *PC1* reflects a polarization between OxPhos (positive, *magenta*) to mitochondrial central dogma-related pathways (negative, *orange*). *PC2* reflects a polarization between a mixed set of pathways (positive) to metabolism-related pathways (negative, *teal*). *PC3* reflects a polarization between metabolism-related pathways (positive, *teal*) to a mixed set of pathways including OxPhos and mitochondrial central dogma (negative, *magenta* and *orange*, respectively).

This mitoPPS analysis offers some interesting findings. The brain and immune mitotypes occupy the most extreme contrasting positions on PC1 (−0.3 vs +0.2) but have similar PC2 values (0.1 vs 0.2), reflecting the prioritization of *OxPhos* in brain mitochondria, and of *Proteases* and *mtDNA-related central dogma-*related pathways in immune mitochondria. In contrast, both tissues similarly deprioritize metabolism-related pathways such as amino acid metabolism and fatty acid oxidation, which are selectively prioritized by the liver. In contrast, the liver sits at similar PC1 (0.08 vs 0.19) and PC3 (0.05 vs −0.01) coordinates as the brain, but diverge most strongly on PC2 (−0.50 vs 0.10) reflecting the prioritization of *Lipid* and *Amino acid metabolism,* and the de-prioritization of *Autophagy* and *mtDNA gene expression* in liver mitochondria.

This mitoPPS-based analysis also clarifies what makes muscle mitochondria most distinct from other tissues. The skeletal muscle and heart mitotype segregates away from all other tissues along PC3, illustrating how they prioritize iron-sulfur containing proteins, the cristae-remodeling MICOS complex, and CV assembly factors.

Figure 5F illustrates the prioritization for 10 of the most widely studied mitochondrial pathways across 8 vital organs. The adrenal gland mitotypes prioritize *Cholesterol, bile acid, and steroid synthesis* pathways (14.6-fold over other tissues) consistent with their primary role not in energy transformation, but in steroidogenesis (adrenal mitochondria are the sole site of all steroid hormone synthesis ^41^). The co-regulation of multiple mitoPPS across organ systems, emphasized in the vertical dendrogram of Figure 5A (more details in **Supplemental Figure S6A**) illustrates how adrenal gland mitochondria also specialize in complimentary endocrine pathways, such as *Vitamin D metabolism* (19.4-fold), and to a lower extent in other pathways required for redox-based biosynthetic reactions (e.g. Fe-S biosynthesis, 1.85-fold). The liver, on the other hand, prioritizes CII and FAO 11.8–25.8-times more than the G3P shuttle respectively, which as noted earlier is strongly deprioritized in liver mitochondria.

### Mitochondrial plasticity in aging human fibroblasts

Many of the mitotype differences described above are likely driven by robust differences in cell-type specific metabolic, biosynthetic and signaling requirements inherent to organismal physiology in animals ^2^. Therefore, to understand to what extent mitochondrial phenotypes can exhibit plasticity *in a given cell type*, we used a controlled *in vitro* monoculture system of primary human dermal fibroblast. The dataset includes RNAseq data from multiple donors (n=8) exposed to pharmacological, metabolic, and endocrine challenges (n=13 treatment conditions), longitudinally monitored at intervals of 12–20 days for up to 9 months (40 passages), representing a total of 339 datapoints: the *Cellular Lifespan Study* ^6^.

Compared to the GTEx dataset that examines mitotypes from different organs in their normal physiological states regulated by organismal cues, this system allows to ask in a more definitive manner which MitoPathways are necessarily (i.e., genetically) co-regulated, and which subsets of mitochondrial functions can be uncoupled during stressful conditions or with aging.

**Figure 6:**
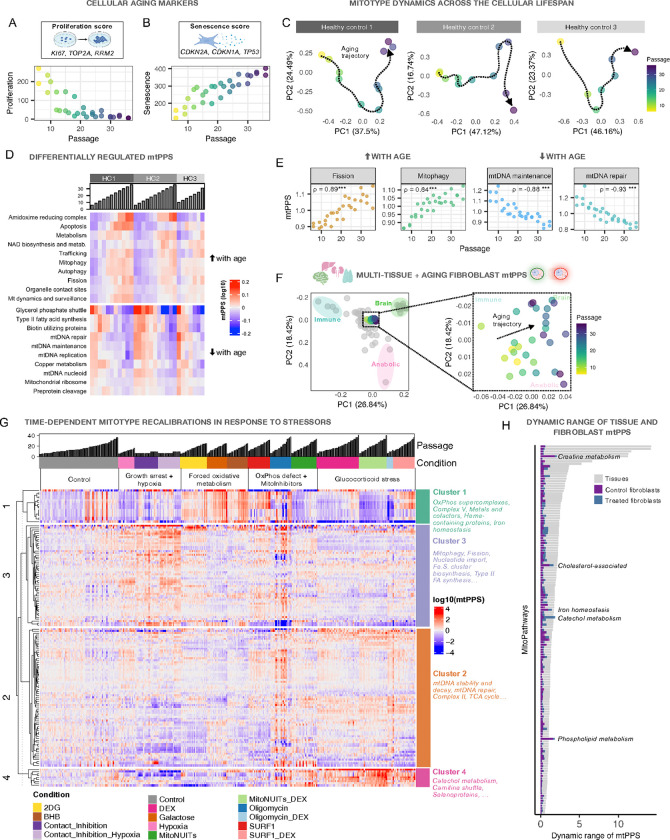
Mitochondria dynamically recalibrate in response to experimental perturbations. **A)** The proliferation index (average expression of genes *KI67, TOP2A, and RRM2*) is downregulated and **B)** the senescence index (*CDKN2A, CDKN1A, and TP53*) is upregulated in healthy control fibroblasts cultured over 30+ passages. **C)**Principal component analysis of MitoPathway mitoPPS from three healthy controls. Dotted lines indiciate individual aging trajectories, suggesting a gradual MitoPathway priority shift with cellular age. **D)**Heatmap of top ten up – and downregulated mitoPPS (log10) with age in three healthy controls. Columns represent samples sorted by cell line and ascending passage. **E)** Scatter plot and spearman’s r of two pathways that become prioritized (Fission, Mitophagy, *left panel*) and deprioritized (mtDNA maintenance, mtDNA repair, *right panel*) with age. **F)**PCA of the multi-tissue Human Protein Atlas mitoPPS with the immune, brain, and anabolic axes, and Cellular Lifespan Study mitoPPS (left panel). Each dot represents one tissue (grey) or fibroblast sample from healthy controls (color gradient by passage). The right panel shows the same PCA as in the left panel, highlighting the fibroblast samples. **G)**Heatmap of mitochondrial recalibrations in response to experimental perturbation. Rows represent MitoPathways and columns represent samples, sorted by experimental condition and ascending passages. **H)**Dynamic range of tissue and fibroblast mitoPPS. For tissues, the dynamic range was calculated across 55 human tissues using the delta between the lowest and highest mitoPPS. For control fibroblasts, the dynamic range was determined along the lifespan trajectories show in Figure 6C from 3 cell lines. The treatment fibroblast dataset was first averaged across cell lines and conditions to remove the aging effect, and the dynamic range was determined using the delta mitoPPS from lowest to highest.

To interrogate how mitotype profiling can help understand dynamic mitochondrial recalibrations, we focused our mitoPPS analysis on the temporal changes in untreated cells from the three most deeply phenotyped donors (Health Control, HC1–3). We previously confirmed that time in culture or the number of passages in this model are associated with canonical hallmarks of aging including progressive telomere shortening, increased epigenetic clocks, and increased expression of senescence markers ^7,8^. Figures 6A,B show RNA levels for indices of senescence (CDKN2A, CDKN1A, TP53) and proliferation markers (KI67, TOP2A, RRM2), confirming the robust expression of late-life transcriptional programs. This provides an ideal setting to examine how mitochondria quantitatively recalibrate as cells initiate aging programs.

#### Mitotype shifts across cellular aging

The mitotype signature (i.e. mitoPPS only, no canonical age-related genes) of all timepoints for each donor projected in a PCA space revealed that mitochondrial aging follows at least a two-phase pattern (Figure 6C). For example, the mitotype of HC1 initially goes down (along PC2) and right (along PC1) over the first 21 passages (~100 days of culture), and climbs gradually on PC2 while remaining relatively constant on PC1 in later life. Interestingly, each donor exhibited a relatively unique trajectory, possibly mirroring inter-individual heterogeneity in human cellular aging ^42^. The pathways loading most strongly on each PC for each donor are listed in Supplemental File 2. We then asked which mitoPPS changed most consistently with increasing cellular age. The top 10 most upregulated and downregulated pathways are visualized in Figure 6D. Among the most upregulated pathways were mitochondrial fission and mitophagy involved in mitochondrial quality control with aging (Figure 6E). In contrast, among the most downregulated pathways were mtDNA related pathways, such as maintenance and repair, the same pathways associated with immune mitotypes (see PCA loadings in Figure 5E).

This finding inspired us to explore whether mitoPPS from the longitudinal fibroblast dataset could be integrated into the multi-tissue dataset. This integration is possible given that both mitoPPS datasets are unitless and internally normalized to other MitoPathways. As expected, the fibroblast mitoPPS clustered in the center of the tissue distribution near the skin and other epithelial tissue mitotypes, suggesting their similar mitotype signature (Figure 6F). In relation to aging cells, the fibroblast mitoPPS time trajectories projected in the multi-tissue space showed that aging mitochondria transition along PC1 away from immune tissues (which upregulate mtDNA-related pathways) towards the more catabolic brain phenotype. Further supporting this observation, OxPhos supercomplex-related mitoPPS (upregulated in brain mitotypes) increased with aging in healthy, untreated cells (Figure 6G left side of the heatmap). At a high level, this pattern across the cellular lifespan may indicate a shift in priority from a proliferative/signaling mitotype (immune tissues) towards a less versatile, energy transforming “senescent” mitotype. These findings demonstrate the application of mitoPPS-based mitotyping from RNAseq in cultured cells, and its integrability with a multi-tissue human dataset.

### Mitotype recalibrations to targeted metabolic perturbations

Finally, we examined how much plasticity the human fibroblast mitotype can exhibit in response to various metabolic perturbations. In addition to the serial passages modeling the aging process, the *Cellular Lifespan Study* dataset includes robust experimental perturbations: OxPhos inhibition with low dose (1uM) oligomycin, genetic mutations in the SURF1 genes that impairs basal OxPhos and upregulates glycolysis ^6^, hypoxia (3% O_2_ compared to ambient 21%), and long-term growth arrest (up to 140 days in contact inhibition without passaging). Metabolic manipulations that influence mitochondrial carbon substrates availability included: i) galactose without glucose or ii) 2-deoxyglucose (2-DG) that directly inhibits glycolysis; iii) 2-beta-hydroxybutyrate (BHB), a ketone body oxidized in mitochondria; and iv) a combination of three mitochondrial nutrient uptake inhibitors (NUITs: UK5099, Etomoxir, BPTES) that block mitochondrial pyruvate, medium- and long-chain fatty acid, and glutamine transport, starving the Krebs cycle of carbon substrates (see ^6^ for details). Together, these chronic experimental conditions applied for up to 9 months constitute a robust test whether and how much the mitotype of a given cell type can recalibrate.

For each timepoint and cell line, we calculated mitoPPS relative to controls across all treatment conditions. The mitoPPS scores are visualized in Figure 6G (see **Supplemental Figure S8** for details). As for the aging fibroblast, the multi-treatment data was integrated into the multi-tissue mitoPPS dataset (**Supplemental Figure S8D,E**). The overall mitochondrial specialization profiles show that various treatment combinations have coordinated effects on multiple MitoPathways. For example, the glycolysis inhibitors 2-DG, Galactose, and BHB forced an expected prioritization of OxPhos-related pathways (Cluster 1 in Figure 6G) and occupy a similar PCA space shifted towards the highly oxidative and catabolic brain mitotype. Relative to all treatment conditions, Galactose-treated fibroblasts prioritized Complex I by +18%, while the cerebral cortex prioritized Complex I by +163% relative to all other tissues (**Supplemental Figure S9A**). On the other hand, cells treated with the synthetic glucocorticoid Dexamethasone and MitoInhibitors combined show a shift towards the anabolic mitotype along PC2. Indeed, those cells show the greatest but subtle shift towards FAO (+11–13%). In comparison the liver prioritizes FAO by +216% (**Supplemental Figure S9B**). These results confirm the expected patterns of mitochondrial specialization by each treatment, and illustrate their smaller magnitude than that observed across human tissues.

Applying the same quantitative framework, we show that aging cells deprioritize mtDNA repair by up to −35% from youngest to oldest passages, while immune tissues prioritize mtDNA repair by +75% (tonsil) to +125% (bone marrow) relative to all other tissues (**Supplemental Figure S9C**). These effects were remarkably reliable across all cell lines.

An important, consistent observation is that the effect sizes for fibroblasts mitotype specialization were small compared to those among tissues. The dynamic range in MitoPPS scores are quantified for *in vitro* fibroblasts and human tissues in Fig 6H. Nevertheless, we note that some MitoPathways exhibited large shifts in response to certain treatments, yielding dynamic ranges comparable or even greater in magnitude than the exceptional inter-tissue diversity (**Supplemental Figure S10B,C**). Thus, even in a single cell type, mitochondria exhibit highly dynamic and plastic phenotypes quantified by their MitoPPS.

## Discussion

Tissues and cell types have the same genetic hardware but run specialized transcriptional programs that subserve specialized metabolic needs, altogether sustaining organismal life. To capture the remarkable diversity and plasticity of mitochondrial phenotypes, we developed a simple computational approach that extracts mitochondrial pathway prioritization scores (MitoPPS) from transcriptomics data. By deploying the mitotyping pipeline to systematically and quantitatively define mitochondrial phenotypes (i.e., mitotypes) across mouse and human tissues, as well as in cultured human fibroblasts, we show how much mitochondria from the same organism and cell type can adopt different molecular profiles, and how much these can change over time and in response to metabolic challenges. The mitotyping approach accessible at MitotypeExplorer.org and in the associated code equips non-experts and experts with a portable approach to navigate and interpret the multifaceted, dynamic nature of mitochondria.

The mitotyping approach differs from traditional gene set enrichment analyses in several ways. In particular, it includes a detailed curated inventory of interpretable mitochondrial functions (e.g., TCA cycle, anaplerosis) rather than more general labels and compartments (e.g., mitochondrial cristae or matrix). Our findings raise the question whether some observed molecular signatures reflect stable mitotypes or dynamic mitochondrial states – similar to transient “activated”, “progenitor”, “differentiated”, “aged”, and other cellular states ^11,12^. In tissues as in cells, mitotypes likely reflect a combination of both, especially given that different mitotypes can exist within distinct sub-compartments of the same cell ^1,20,43^. Developing single cell proteomic and transcriptomic technologies could enable to track dynamic changes in mitochondrial states more sensitively and in a cell-type specific manner. Technically, compared to the simple MitoPathway scores (Figures 3+4), the mitoPPS approach offers a more sensitive approach to quantify specialization across tissues and samples since it is not confounded by overall mitochondrial expression and can more easily be integrated across datasets.

Some limitations should be noted. First, our tissue-based analyses reflect a mix of different cells and is subject to tissue heterogeneity variations. Extending Mitotyping at the single-cell level ^44^ should allow to harness forthcoming resources such as the Human Cell Atlas ^45^ to address more specific questions in different cell (sub)types. Nevertheless, it will remain difficult even at that level to disentangle cell-type-specific mitochondrial phenotypes from mitotypes, as highly dynamic mitochondrial states cannot be mapped with current proteomic and RNAseq-based technologies. MitoPPS-based enrichment reflects the activation of cellular programs (i.e., what the cell is attempting to accomplish) rather than the actual functional capacity of its mitochondrial population. For instance, both fusion and fission MitoPathways are typically strongly correlated, likely reflecting the potential or propensity of mitochondria to undergo fusion and fission dynamics in response to stimuli, rather than the actual occurrence of fusion and fission events or the morphological state of the mitochondrial network. Future studies could utilize paired RNAseq, proteomics, and other modalities such as respirometry or fluxomics to map the temporal relationship between the initial shift in a transcriptional mitotype and the effective changes at the protein and functional levels. Mitotype shifts also could occur independent of transcription. In relation to our *in vitro* results, our findings reflect not human aging but cellular aging in a replicative (i.e., Hayflick-style) lifespan model. Finally, we note that a minority of MitoPathways are represented by few genes, which likely decreases the robustness of their mitoPPS estimates. Using MitotypeExplorer.org, the relative contribution of each gene to a given mitoPPS score, the effect of normalization methods, and other features can be explored across mouse and human tissues online. Our quantitative, fine-grained, scalable approach to profiling mitotypes gives researchers the ability to quantify and interpret the multivariate space of mitochondrial biology.

## Methods

### Data sources

This study uses multiple publicly available datasets: Both human and mouse MitoCarta3.0 datasets have been obtained from the Mootha lab at Broad Institute ^5^.

The human protein atlas tissue consensus RNA dataset (used throughout the manuscript) is obtained from the Human Protein Atlas (HPA) resource, and is a combination of transcriptomics data from HPA and GTEx in which the normalized expression value (“nTPM”, used to calculate pathway scores) is the maximum nTPM value for each gene from the two datasets. The resulting data contains one value per gene and tissue.

The GTEx liver and cerebral cortex dataset used in Figure 2F–I and Supplemental Figures S2 C-D and S3 is obtained from the GTEx consortium with 948 total donors and 17382 samples, of which 226 were liver donors, and 255 cortex donors.

The cellular lifespan dataset was obtained from previous work (Sturm 2022).

### Data pre-processing

#### Mouse MitoCarta3.0

The mouse MitoCarta3.0 proteomics dataset of 14 tissues was used as is (total peak intensity). The dataset misses many proteins that are either tissue specific, show different temporal expression patterns, are part of the mitochondrial outer membrane ^5^, or we reasoned they are below the detection limit. Since missing values are problematic for hierarchical clustering and principal component analysis, we imputed some, yet not all, missing values. First, we removed proteins that were not detected in any of the 14 tissues (163 proteins). For the remaining proteins, we reversed the log10 transformed data, and imputed NAs as half of the minimal value of each gene to get a low value that is non-zero. In total, 3729 (27%) missing values were imputed, resulting in a total of 977 proteins in 14 tissues. We next assigned groups to tissues with functional similarities (**Supplemental table 1**).

#### Human MitoCarta3.0

The human MitoCarta3.0 dataset served as both, a database of mitochondrial genes (both nuclearDNA- and mitochondrialDNA-endcoded, 1136 genes), as well as a resource for gene-to-pathway annotations (Supplemental File 1). Some genes play a role in multiple pathways, and the amount of genes within each pathway differ (see Supplemental table 2). Pathways exist on three levels that we outline here with OxPhos as example: the first level pathway “OxPhos”, contains multiple second level pathways (“Complex I”, “Complex II”,…), and multiple third level pathways (“Complex I subunits”, “Complex I assembly factors”). The latter two usually have no shared genes, but level 2 pathways contain all level 3 genes, and level1 pathways contain genes of levels 2 and 3. Thus, some pathways can show similar expression patterns due to shared genes.

#### Human Protein Atlas multi-tissue dataset

The Human Protein Atlas consensus tissue transcript expression dataset, based on transcriptomics data from HPA and GTEx ^29–31^, was accessed at version 21.0 (2021.11.18) with ENSEMBL version 103.38. The data format was unchanged (nTPM = normalized transcripts per million, TMM normalization and 1134 mitochondrial genes in 55 human tissues were found. One gene was missing (RP11_469A15.2) and one gene (TSTD3) was excluded due to missing values in multiple tissues. Same as for the mouse proteomics dataset, we assigned groups to tissues with functional similarities as shown in Supplemental table 1. Any modification to the nTPM data (zscore-transform, log10 transform) is described in the respective figure legend. Given that the HPA dataset contains *one* value per tissue (summarized across two datasets from multiple donors), statistical power is limited. Multi-donor datasets such as the Genotype-Tissue Expression (GTEx) should be explored in future studies for robustness and to explore inter-individual variability. Another limitation of this (bulk transcriptomics) dataset is that the data is composed of cell type mixtures, likely with one dominating cell type (for example cardiomyocytes in the heart). As a result, such tissue-based analyses may underestimate the true magnitude of differences between pure mitotypes. Resources such as the Human Cell Atlas ^45^ can further our understanding of cell-type level mitotypes.

#### GTEx multi-individual dataset

We used GTEx version 8 (2017-06-05) ^31,46^ raw count data (gene_reads) and applied TMM normalization using the edgeR package ^47^ . Although the data can be accessed as transcripts per million (TPM), we reasoned that varying library sizes (i.e. the total number of mapped gene reads) can lead to over- or underestimation of MitoPathway scores. We demonstrate the differences in library sizes using four distinct tissues (brain – cortex, liver, skeletal muscle, and whole blood, **Supplemental Figure S3**). Library sizes within tissues can vary by 13-fold, and whole blood library sizes are generally lower compared to the other three tissues. This is most likely due to lower amounts of mtDNA-encoded transcripts and fewer copies thereof, further confirming the necessity to correct for it ^48,49^. We next compared the effect of normalization using histograms for each of the four tissues and MitoPathway scores of the three pathways studied in GTEx (Complex I, Fatty acid oxidation, and Complex II). While the directionality between the uncorrected (TPM) and library-corrected (TMM) pathway scores is largely similar, the data distribution within tissue groups varies between the two methods. For example, Complex II expression is low in brain and whole blood, compared to liver and muscle. While the whole blood Complex II TPM score is skewed towards lower expression, TMM normalization corrects this towards a normal distribution, while brain and whole blood still remain lowest.

After TMM normalization, we pulled all mitochondrial genes from the GTEx dataset using ENSEMBL mapping between MitoCarta and GTEx (see code for details). In total, we identified 1133 genes in 54 tissues and 928 donors. The three missing genes were SOC2, CMC4, and ATP5MF-PTCD1. For GTEx mitotyping (Figure 2 F–I) we used only data from the cerebral cortex (n=255 donors) and liver (n=226 donors), among which 79 cortex and liver samples were from the same donor.

#### Cellular lifespan study

The unprocessed cellular lifespan dataset (GSE179848) can be accessed via Gene Expression Omnibus (GEO. The data was imported using txi import (length-scaled TPM), and further TMM normalized using the NOISeq package. The resulting nTPM values are the TMM normalized protein-coding transcripts per million data. 1135 mitochondrial genes were found (RP11_469A15.2 missing). We noticed that mtDNA-encoded genes such as MT-ND1 and MT-ND2 were missing the “MT-“ suffix, which was added manually. In addition, gene C12orf10 was renamed to its synonym MYG1 for mapping. One sample (RNAseq_Sample152, part of Figure 6H) was excluded due to infinity values in MitoPathway scores (see code for details). During the course of the experiments, we noticed that one (apparently healthy) control line used (HC3, Coriell AG01439) was likely isolated from a diseased newborn, since the infant had died of unknown cause 4 days after birth ^8^. In total, 344 samples from 7 cell lines and 15 conditions (genetic differences or treatments or both) were mitotyped.

### Quantification and Statistical analyses

#### Mouse FAO score

A fatty acid oxidation score was calculated using expression data from the following genes: Acaa1a, Acaa2, Acacb, Acad10, Acad11, Acad12, Acadl, Acadm, Acads, Acadsb, Acadvl, Acat1, Acot11, Acsf2, Acsl1, Acsl6, Acsm1, Acsm2, Acsm3, Acsm4, Acsm5, Acss1, Amacr, Cpt1a, Cpt1b, Cpt1c, Cpt2, Crat, Crot, Decr1, Echs1, Eci1, Eci2, Etfb, Etfdh, Hadh, Hadha, Hadhb, Hsd17b10, Mcee, Mmut, Pcca, Pccb, Slc25a20. *A* score was calculated using the average normalized expression of the aforementioned genes.

#### Mitopathway scores

As mentioned above, gene-to-pathway annotations were extracted from MitoCarta3.0. To calculate MitoPathway scores from any normalized expression data (HPA, GTEx, Cellular lifespan study), expression values of all genes within a given pathway were averaged. Pathways that contain mtDNA-encoded genes always rank highest due to multiple copies of the same gene. Hence, MitoPathway scores should be expressed in *relative* terms (e.g. relative to all other tissues, e.g. which tissue ranks highest in MitoPathway Complex I). Importantly, the scoring requires that most mitochondrial genes associated with a given pathway are represented in the dataset. Insufficient gene coverage can affect the reliability and biological relevance of the calculated scores, potentially leading to inaccurate interpretations.

Any modification to the data (zscore, log10 transform, log2 fold changes, ratios) is specified in figure legends. Typically, data was zscore-transformed within pathways, log2 fold changes were calculated between two tissues and ratios were calculated between two pathways for each tissue.

#### mitoPPS

##### Human Protein Atlas mitoPPS

Given that MitoPathway scores differ by multiple orders of magnitude (e.g. complex I scores spans 5 orders of magnitude, while fatty acid oxidation scores spans 2 orders of magnitude from lowest to highest tissue), they cannot be expressed and compared in absolute terms. In addition, tissues with a naturally greater mitochondrial content rank highest in most MitoPathways. To address this scaling issue, we used a normalized ratio-based approach that determines how much a sample prioritizes a MitoPathway over all other MitoPathways and samples. We eliminated the effect of overall mitochondrial gene expression level by dividing individual MitoPathway score ratios by the average ratios across all samples in the dataset for each respective pathway pair. Thus, for each sample of interest $S_{i}$ and pathway of interest $P_{i}$ the respective mitoPPS is calculated as follows:

$$mitoPPS_{S_{i}P_{i}}\left(n^{\prime },x^{\prime }\right)=\frac{1}{n^{\prime }-1}\sum_{\begin{array}{c} n\geq 1\\ Pn\neq PI \end{array}}^{n\prime -1}\;\frac{{Score}_{S_{i}P_{i}}\;\times \;{\left({Score}_{S_{i}P_{n}}\right)}^{-1}}{\frac{1}{x^{\prime }}\sum_{x\geq 1}^{x\prime }{Score}_{S_{x}P_{i}}\;\times \;{\left({Score}_{S_{x}P_{n}}\right)}^{-1}}$$

Where

$S_{i}$ = *Sample of interest*

$P_{i}$ = *Pathway of interest*

Score = *MitoPathway score*

$n^{\prime }$ = *Total number of Pathways*

$x^{\prime }$ = *Total number of Samples*

$P_{n}=n^{\mathrm{th}}$ *pathway in set of pathways P excluding PI*

$S_{x}=X^{\mathrm{th}}$ *sample in a dataset*

##### Cellular lifespan study mitoPPS

We used the same pipeline as outlined above for the HPA dataset, but used the average ratio of controls (untreated and healthy) as reference for the correction. For Figure 6B–G we used control samples from the longest culture from study part 2 ^6^, but included all data in the heatmap in Figure 6H.

#### Hierarchical clustering + PCA

All heatmaps were generated using the ComplexHeatmap package ^50,51^. Data transformations (z-score, log transform) can be found in the figure legends. For hierarchical clustering, the Euclidean distance was calculated, and cluster analysis was performed using the Ward’s D2 hierarchical Agglomerative Clustering Method ^52^. If a different method (such as k-mean clustering) was used, the information can be found in the figure legends.

Principal component analysis was conducted using the base R prcomp function. Loadings can be found in the supplemental information. 3D plots of the principal components were generated using the rgl package and the plot3d function.

#### Radar charts

Radar charts were generated from z-score transformed (MitoPathwayScores) or mitoPPS data using the fmsb package. The boundaries of the radar chart are scaled within each dataset, with 100% representing the highest value (+10%) and 0% representing the lowest value (−10%).

#### Statistical tests

All statistical tests relevant to the figures can be found in the figure legends. Statistics not reported in the figures (but reported in the text) can be found in the associated code on GitHub. All correlations in this manuscript were performed using Spearman’s rank-order correlation with the cor.test function (stats package). Effect size was calculated using Cohen’s D with Hedges’ g correction using the cohen.d function from the effsize package (hedges.correction=TRUE). When two groups were directly compared, first a Shapiro-Wilk test (stats package) was used to test for normal distribution, and a Fliegner-Killeen test (stats package) was used to test for equal variance. If the data was normally distributed and variances were homogeneous, a Student’s t-Test (stats package) was performed. If the assumption of equal variance was not met in a normally distributed set of data, a Welch’s t-Test was performed. Non-parametric tests were used when the data was not normally distributed. For homogeneous variance a Wilcoxon Rank Sum test (Mann Whitney, stats package) was performed. If the variance was not homogeneous, a Brunner-Munzel test (brunnermunzel package) was used.

## Data Availability

All datasets used in this study are publicly available and can be accessed on the respective domain. The code is written in R version 4.3.2 (2023–10-31), and can be accessed through GitHub, together with the package versions and the processed (i.e. “mitotyped”) data used to create the figures. (https://github.com/annamonzel/mitotyping)

## References

[1] Pekkurnaz G. & Wang X. Mitochondrial heterogeneity and homeostasis through the lens of a neuron. Nat Metab, doi:10.1038/s42255-022-00594-w (2022). PMC11151822 35817853

[2] Picard M. & Shirihai O. S. Mitochondrial signal transduction. Cell Metab 34, 1620–1653, doi:10.1016/j.cmet.2022.10.008 (2022). 36323233 PMC9692202

[3] Ryu K. W. Cellular ATP demand creates metabolically distinct subpopulations of mitochondria. Nature 635, 746–754, doi:10.1038/s41586-024-08146-w (2024). 39506109 PMC11869630

[4] Monzel A. S., Enríquez J. A. & Picard M. Multifaceted mitochondria: moving mitochondrial science beyond function and dysfunction. Nature Metabolism 5, 546–562, doi:10.1038/s42255-023-00783-1 (2023). PMC10427836 37100996

[5] Rath S. MitoCarta3.0: an updated mitochondrial proteome now with sub-organelle localization and pathway annotations. Nucleic Acids Res 49, D1541–D1547, doi:10.1093/nar/gkaa1011 (2021). 33174596 PMC7778944

[6] Sturm G. A multi-omics longitudinal aging dataset in primary human fibroblasts with mitochondrial perturbations. Sci. Data 9, 751, doi:10.1038/s41597-022-01852-y (2022). 36463290 PMC9719499

[7] Sturm G. OxPhos defects cause hypermetabolism and reduce lifespan in cells and in patients with mitochondrial diseases. Commun. Biol. 6, 22, doi:10.1038/s42003-022-04303-x (2023). 36635485 PMC9837150

[8] Bobba-Alves N. Cellular allostatic load is linked to increased energy expenditure and accelerated biological aging. Psychoneuroendocrinology 155, 106322, doi:10.1016/j.psyneuen.2023.106322 (2023). 37423094 PMC10528419

[9] Marieb E. & Hoehn K. Essentials of Human Anatomy & Physiology. (Pearson Edication Limited, 2022).

[10] Arendt D. The evolution of cell types in animals: emerging principles from molecular studies. Nat. Rev. Genet. 9, 868–882, doi:10.1038/nrg2416 (2008). 18927580

[11] Zeng H. What is a cell type and how to define it? Cell 185, 2739–2755, doi:10.1016/j.cell.2022.06.031 (2022). 35868277 PMC9342916

[12] Xia B., Yanai I., Klein A. & Treutlein B. A periodic table of cell types. Development 146, dev169854, doi:10.1242/dev.169854 (2019). 31249003 PMC6602355

[13] Green G. S. Cellular communities reveal trajectories of brain ageing and Alzheimer’s disease. Nature 633, 634–645, doi:10.1038/s41586-024-07871-6 (2024). 39198642 PMC11877878

[14] Suomalainen A. & Nunnari J. Mitochondria at the crossroads of health and disease. Cell, 2601–2627, doi:10.1016/j.cell.2024.04.037 (2024). 38788685

[15] Rodriguez-Nuevo A. Oocytes maintain ROS-free mitochondrial metabolism by suppressing complex I. Nature, doi:10.1038/s41586-022-04979-5 (2022). PMC9329100 35859172

[16] 16 Pagliarini D. J. A mitochondrial protein compendium elucidates complex I disease biology. Cell 134, 112–123, doi:10.1016/j.cell.2008.06.016 (2008). 18614015 PMC2778844

[17] Granath-Panelo M. & Kajimura S. Mitochondrial heterogeneity and adaptations to cellular needs. Nat. Cell Biol. 26, 674–686, doi:10.1038/s41556-024-01410-1 (2024). 38755301 PMC12204375

[18] Rausser S. Mitochondrial phenotypes in purified human immune cell subtypes and cell mixtures. eLife 10, doi:10.7554/eLife.70899 (2021). PMC8612706 34698636

[19] Picard M., Hepple R. T. & Burelle Y. Mitochondrial functional specialization in glycolytic and oxidative muscle fibers: tailoring the organelle for optimal function. Am J Physiol Cell Physiol 302, C629–641, doi:10.1152/ajpcell.00368.2011 (2012). 22031602

[20] Benador I. Y. Mitochondria Bound to Lipid Droplets Have Unique Bioenergetics, Composition, and Dynamics that Support Lipid Droplet Expansion. Cell Metab 27, 869–885 e866, doi:10.1016/j.cmet.2018.03.003 (2018). 29617645 PMC5969538

[21] Meinild Lundby A. K. Exercise training increases skeletal muscle mitochondrial volume density by enlargement of existing mitochondria and not de novo biogenesis. Acta Physiol (Oxf) 222, doi:10.1111/apha.12905 (2018). 28580772

[22] Burelle Y. & Hochachka P. W. Endurance training induces muscle-specific changes in mitochondrial function in skinned muscle fibers. J Appl Physiol (1985) 92, 2429–2438, doi:10.1152/japplphysiol.01024.2001 (2002). 12015357

[23] Duking T. Ketogenic diet uncovers differential metabolic plasticity of brain cells. Sci Adv 8, eabo7639, doi:10.1126/sciadv.abo7639 (2022). 36112685 PMC9481126

[24] 24 Du J. Dynamic regulation of mitochondrial function by glucocorticoids. Proc. Natl. Acad. Sci. 106, 3543–3548, doi:10.1073/pnas.0812671106 (2009). 19202080 PMC2637276

[25] Picard M., McEwen B. S., Epel E. S. & Sandi C. An energetic view of stress: Focus on mitochondria. Front Neuroendocrin 49, 72–85, doi:10.1016/j.yfrne.2018.01.001 (2018). PMC5964020 29339091

[26] Picard M. & McEwen B. S. Psychological Stress and Mitochondria. Psychosom. Med. 80, 126–140, doi:10.1097/psy.0000000000000544 (2018). 29389735 PMC5901651

[27] Trumpff C. Psychosocial experiences are associated with human brain mitochondrial biology. Proc Natl Acad Sci U S A 121, e2317673121, doi:10.1073/pnas.2317673121 (2024). 38889126 PMC11228499

[28] Picard M. The mitochondrial phenotype of peripheral muscle in chronic obstructive pulmonary disease: disuse or dysfunction? Am J Respir Crit Care Med 178, 1040–1047, doi:10.1164/rccm.200807-1005OC (2008). 18755922

[29] Karlsson M. A single-cell type transcriptomics map of human tissues. Sci Adv 7, doi:10.1126/sciadv.abh2169 (2021). PMC8318366 34321199

[30] HPA-Portal. (https://www.proteinatlas.org/about/download).

[31] GTEx-Consortium. The GTEx Consortium atlas of genetic regulatory effects across human tissues. Science 369, 1318–1330, doi:10.1126/science.aaz1776 (2020). 32913098 PMC7737656

[32] Menacho C. & Prigione A. Tackling mitochondrial diversity in brain function: from animal models to human brain organoids. Int. J. Biochem. Cell Biol. 123, 105760, doi:10.1016/j.biocel.2020.105760 (2020). 32339638

[33] Morio B., Panthu B., Bassot A. & Rieusset J. Role of mitochondria in liver metabolic health and diseases. Cell Calcium 94, 102336, doi:10.1016/j.ceca.2020.102336 (2021). 33387847

[34] Inigo M., Deja S. & Burgess S. C. Ins and Outs of the TCA Cycle: The Central Role of Anaplerosis. Annu Rev Nutr 41, 19–47, doi:10.1146/annurev-nutr-120420-025558 (2021). 34270333

[35] Bender H. U. Functional consequences of PRODH missense mutations. Am J Hum Genet 76, 409–420, doi:10.1086/428142 (2005). 15662599 PMC1196393

[36] Jang C. Metabolite Exchange between Mammalian Organs Quantified in Pigs. Cell Metab 30, 594–606.e593, doi:10.1016/j.cmet.2019.06.002 (2019). 31257152 PMC6726553

[37] Dhoundiyal A., Goeschl V., Boehm S., Kubista H. & Hotka M. Glycerol-3-Phosphate Shuttle Is a Backup System Securing Metabolic Flexibility in Neurons. J. Neurosci. 42, 7339–7354, doi:10.1523/jneurosci.0193-22.2022 (2022). 35999055 PMC9525167

[38] Cook I., Wang T. & Leyh T. S. Tetrahydrobiopterin regulates monoamine neurotransmitter sulfonation. Proc. Natl. Acad. Sci. 114, E5317–E5324, doi:10.1073/pnas.1704500114 (2017). 28630292 PMC5502633

[39] 39 Yang H. Revisiting the role of serine metabolism in hepatic lipogenesis. Nature Metabolism 5, 760–761, doi:10.1038/s42255-023-00792-0 (2023). 37169876

[40] Quintana A., Kruse S. E., Kapur R. P., Sanz E. & Palmiter R. D. Complex I deficiency due to loss of Ndufs4 in the brain results in progressive encephalopathy resembling Leigh syndrome. Proc Natl Acad Sci U S A 107, 10996–11001, doi:10.1073/pnas.1006214107 (2010). 20534480 PMC2890717

[41] Miller W. L. Role of mitochondria in steroidogenesis. Endocr Dev 20, 1–19, doi:10.1159/000321204 (2011). 21164254

[42] Oh H. S.-H. Organ aging signatures in the plasma proteome track health and disease. Nature 624, 164–172, doi:10.1038/s41586-023-06802-1 (2023). 38057571 PMC10700136

[43] Willingham T. B., Ajayi P. T. & Glancy B. Subcellular Specialization of Mitochondrial Form and Function in Skeletal Muscle Cells. Front Cell Dev Biol 9, 757305, doi:10.3389/fcell.2021.757305 (2021). 34722542 PMC8554132

[44] Mosharov E. V. A Human Brain Map of Mitochondrial Respiratory Capacity and Diversity. bioRxiv, doi:10.1101/2024.03.05.583623 (2024). PMC12770858 40140564

[45] HCA-Portal. https://data.humancellatlas.org.

[46] GTEx-Portal. https://www.gtexportal.org.

[47] Robinson M. D. & Oshlack A. A scaling normalization method for differential expression analysis of RNA-seq data. Genome Biol. 11, R25, doi:10.1186/gb-2010-11-3-r25 (2010). 20196867 PMC2864565

[48] Evans C., Hardin J. & Stoebel D. M. Selecting between-sample RNA-Seq normalization methods from the perspective of their assumptions. Brief. Bioinform. 19, 776–792, doi:10.1093/bib/bbx008 (2017). PMC6171491 28334202

[49] Zhao S., Ye Z. & Stanton R. Misuse of RPKM or TPM normalization when comparing across samples and sequencing protocols. Rna 26, 903–909, doi:10.1261/rna.074922.120 (2020). 32284352 PMC7373998

[50] Gu Z., Eils R. & Schlesner M. Complex heatmaps reveal patterns and correlations in multidimensional genomic data. Bioinformatics 32, 2847–2849, doi:10.1093/bioinformatics/btw313 (2016). 27207943

[51] Gu Z. Complex heatmap visualization. iMeta 1, e43, doi:10.1002/imt2.43 (2022). 38868715 PMC10989952

[52] Murtagh F. & Legendre P. Ward’s Hierarchical Agglomerative Clustering Method: Which Algorithms Implement Ward’s Criterion? Journal of Classification 31, 274–295, doi:10.1007/s00357-014-9161-z (2014).

